# Early Palliative Care in Degos Disease: A Case for Proactive Support in Rare, Life-Limiting Illnesses

**DOI:** 10.7759/cureus.115104

**Published:** 2026-08-24

**Authors:** Kari Williams, Jamie Ellis-Wittenhagen, Tyler Murphy

**Affiliations:** 1 Division of Palliative Medicine, Department of Medicine, Mayo Clinic Arizona, Phoenix, USA

**Keywords:** atrophic papulosis, kohlmeier-degos disease, palliative care, palliative medicine, rare diseases, supportive care

## Abstract

Kohlmeier-Degos disease is one of the most rarely noted diseases in the medical literature. Due to its rarity, many individuals are likely to go undiagnosed or to be diagnosed in more advanced stages. In those individuals with the disease, the initial presentation consists of characteristic skin lesions (porcelain-white macules). These lesions can remain limited to the skin only, which indicates benign disease. Unfortunately, some patients will go on to develop systemic disease, which is life-threatening and can form lesions in the gastrointestinal tract, central nervous system, heart, and lungs. Because of the complexity and aggressive nature of this disease, early palliative medicine involvement could prove beneficial to support patients with symptom management as well as to support patients and their families in navigating difficult clinical decision-making while pursuing treatment. To the best of our knowledge, this is among the first case reports examining the potential benefits of early integration of palliative medicine and its role in not only systemic Degos disease, but also other rare life-limiting illnesses. This case study will highlight the role of the palliative medicine team’s involvement and the importance of a multidisciplinary team in treating those with rare life-threatening diseases.

## Introduction

Malignant atrophic papulosis, also known as Kohlmeier-Degos disease, is a rarely seen and rarely diagnosed syndrome. Known for its unique skin lesions, the skin rash initially consists of small erythematous papules, typically on the trunk or extremities, and rarely involves the scalp, face, palms, or soles [1]. Within weeks, these progress to larger lesions with a central area of white atrophy surrounded by a telangiectatic rim [2]. If the disease remains localized to the skin only, this is typically a benign process.

On the other hand, Degos disease can progress to systemic involvement, which can take place weeks or years after the skin rash appears. The disease can impact multiple systems, including the gastrointestinal tract (50-60% of cases), followed by the central nervous system (20% of cases) [3]. Other systemic involvement can include the eyes, cardiopulmonary, and hepatorenal systems [3]. Unfortunately, the mortality risk of those with systemic involvement varies within the literature, reporting anywhere from 20% to 50% at five years [1], related to complications of bowel perforation, cerebral artery thrombosis, or cerebral hemorrhage [1]. Given the multi-system organ involvement, these patients tend to have a very high symptom burden for which early palliative care involvement at the time of diagnosis could lead to better overall quality of life in the years ahead.

The exact etiology of this disease remains unclear; however, hypotheses include vasculitis, coagulopathy, or dysfunction of endothelial cells [1]. Genetic predisposition is also a factor that has been investigated following reports among family members and throughout generations [1]. Due to unknown mechanisms leading to cellular dysfunction, treatment is limited. Interventions can include antiplatelet/anticoagulation agents, vasodilators, steroids, immunosuppressants, monoclonal antibodies, and plasma exchange, but there is no significant supportive evidence to prove that these are effective [4]. If gastrointestinal involvement is noted, current recommendations include initiation of eculizumab therapy with the addition of treprostinil [5].

Given the uncertainties surrounding treatment related to this complex disease, early palliative care adds an additional support structure for patients and their families as they navigate many of the unknowns surrounding this illness. Like many rare illnesses, a definitive diagnosis is often a drawn-out process, and the path ahead is typically filled with uncertainty surrounding prognosis, limited treatment options, lack of appropriate resources, and countless care challenges [6]. Since patients with rare illnesses tend to visit with numerous providers from various specialties, an early partnership with the palliative medicine specialty can make care feel less fragmented and more cohesive, while providing whole-person care throughout the entire disease course.

Our case discusses a 38-year-old female with widespread systemic involvement from Degos disease who was directly admitted to our academic medical center for initiation of treprostinil for end-stage, refractory disease. Prior to the hospitalization, the patient and her family had no previous encounters with the palliative medicine specialty. In our case, the hospital-based palliative medicine team was utilized during the most advanced stages of illness to aid with symptom management as well as psychosocial and emotional support with complex decision-making in this rarely seen illness. This case specifically highlights the role of the palliative medicine team in those with rare illnesses and makes a case for consideration of integrating palliative medicine early (at the time of a patient's initial diagnosis) instead of integrating services only at the end of life.

## Case presentation

Background history

A 38-year-old female of South Korean descent with no antecedent past medical history received a formal diagnosis of Kohlmeier-Degos disease at an out-of-state academic and research facility. Prior to this official diagnosis, she had noted classic Degos skin lesions on her arms, legs, and trunk for approximately two years. Based on confirmation of neurologic and intestinal involvement, she was initiated immediately on eculizumab, which was standard of care for systemic disease involvement.

About six months after initiating eculizumab, the patient required an exploratory laparotomy and small bowel resection at a local medical facility due to evidence of intestinal obstruction. Her surgical pathology revealed the presence of extensive Degos plaques within her small and large intestine and throughout the peritoneum. Over the next several months, further complications arose as her disease continued to progress. The patient experienced both abdominal abscesses and recurrent intestinal perforations, necessitating placement of several chronic abdominal drains and dependence on total parenteral nutrition (TPN) therapy.

While hospitalized for the aforementioned issues, she also experienced cardiopulmonary complications, including the development of atrial fibrillation and pleural effusions. In addition, calcifications were noted involving her ureter, leading to nephrostomy tube placement. Throughout these surgeries and interventions, she and her family were never introduced to palliative care services, despite experiencing many difficult-to-control symptoms and complex medical decisions as she faced each repeated surgery and procedure.

Ultimately, several months after her initial bowel surgery, she continued with progressive disease while on eculizumab. Nine months following her formal diagnosis, she eventually sought care at our quaternary academic medical center for consideration of treprostinil therapy for refractory disease. Prior to her elective inpatient admission for the initiation of treprostinil, she had already established care with multiple specialties within our institution, including dermatology, rheumatology, urology, pulmonology, cardiology (advanced heart failure), and cardiothoracic surgery.

Admission to the quaternary care facility

At the time of her hospital admission, treprostinil was started on arrival, and she underwent a planned pleural biopsy with talc pleurodesis due to the ongoing presence of recurrent pleural effusions. Her pathology demonstrated lymphoid infiltrates with mixed benign B and T-cell infiltrates, likely reflective of ongoing vaso-occlusive injury from Degos disease. Following the surgery, she unfortunately developed persistent, uncontrolled pain, and initially, the pain anesthesia service was consulted for pain management. She was placed on a hydromorphone patient-controlled analgesia (PCA) with a recommended increase in her chronic home fentanyl patch. She was additionally started on gabapentin, methocarbamol, and scheduled Tylenol.

Ongoing complications

**Figure 1:**
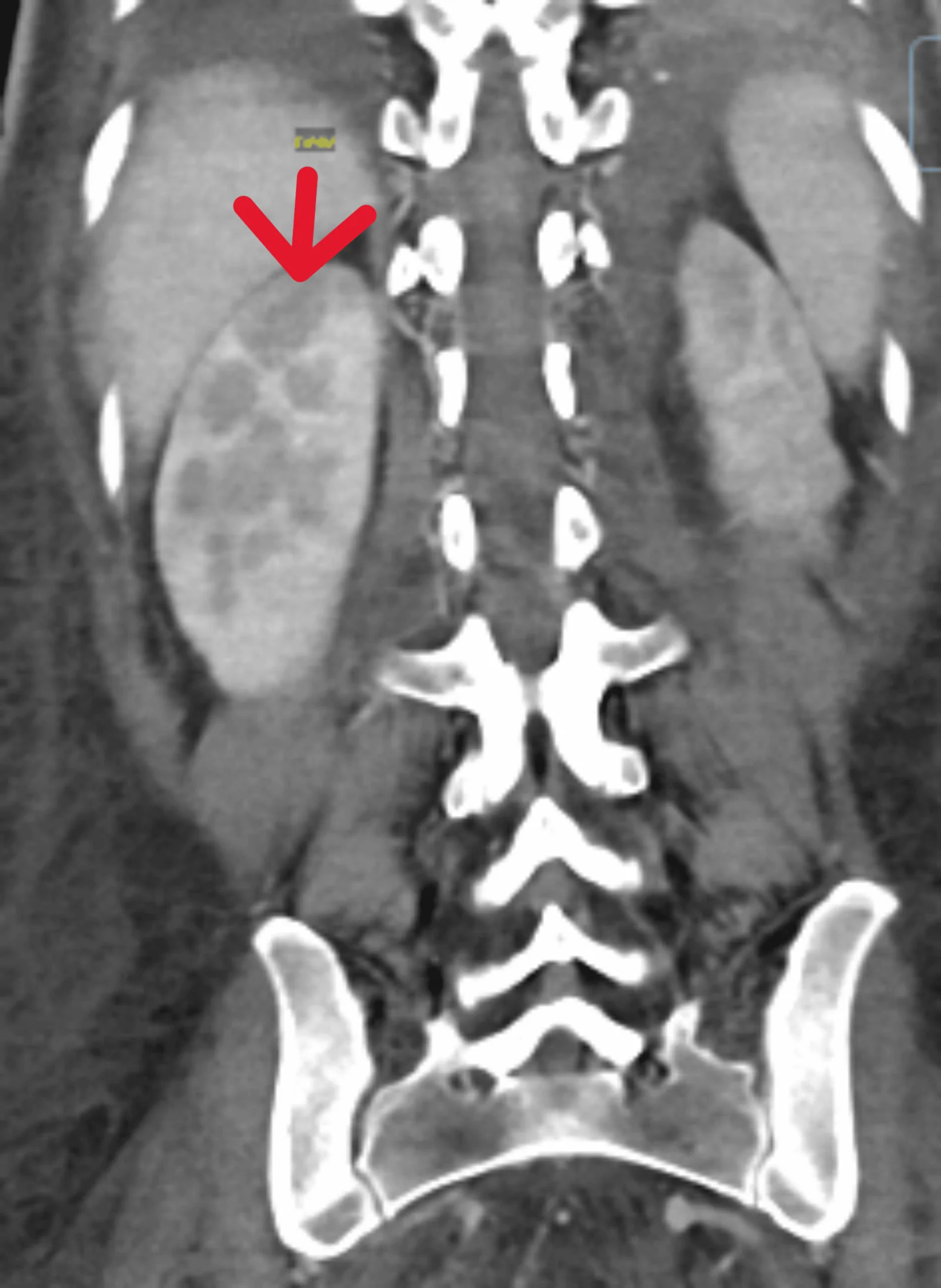
Wedge-shaped hypoenhancement of the right kidney was noted when the patient developed new leukocytosis on day 14 of her hospital stay. This was felt to represent ischemia from vaso-occlusion related to progression of Degos disease.

Over the course of the next two weeks (prior to palliative medicine involvement), her hospital course was plagued with ongoing complications, including development of an ileus, requiring nasogastric tube placement, and development of an enterocutaneous fistula, requiring upsize of her chronic abdominal drain. In addition, a new leukocytosis developed, and CT imaging revealed pyelonephritis with a wedge-shaped enhancement of her right kidney (Figure 1). This was believed to represent ischemia related to another vaso-occlusive event from her underlying Degos disease. Infectious disease consult was completed, and she was started on broad-spectrum antibiotics and antifungal agents.

**Figure 2:**
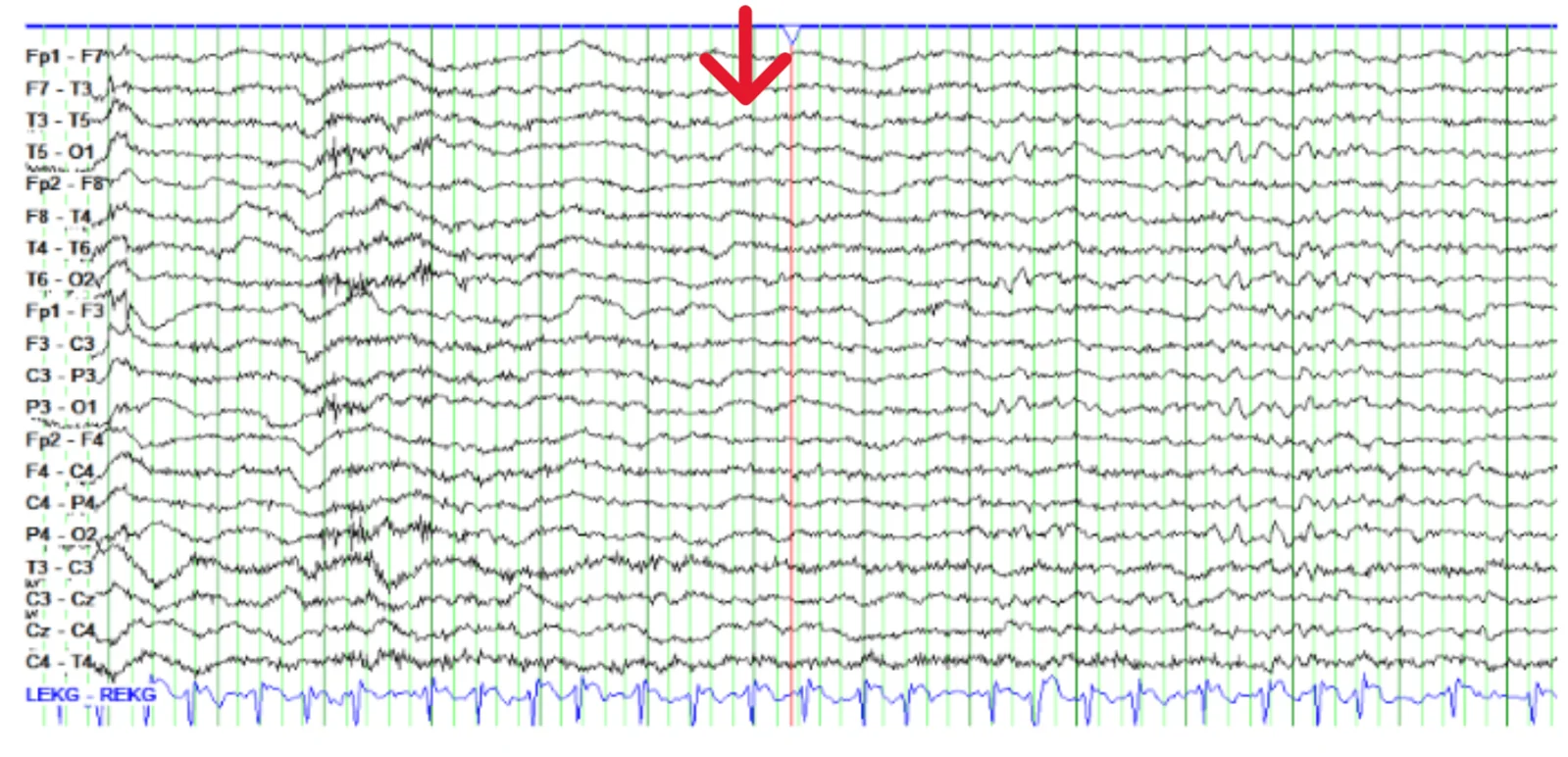
EEG illustrating moderate diffuse slowing. Neurology identified diffuse cortical dysfunction, likely from medications in combination with potential systemic involvement/neurodegenerative findings from her Degos disease.

In conjunction with the above events, she also developed waxing and waning cognition, and an EEG was performed showing generalized slowing consistent with a multifactorial encephalopathy likely related to metabolic derangements and polypharmacy (Figure 2).

Involvement of the palliative medicine team

Ultimately, on day 15 of the hospital stay, the patient developed acute hypoxemic respiratory failure related to ongoing bilateral pleural effusions and potential pneumonitis related to previous use of eculizumab. At this time, the palliative medicine team was formally consulted to assist with symptom management for pain and dyspnea and to also broach goals of care with the patient and family.

At the time of the initial palliative medicine consultation, the patient's symptoms of breathlessness and increased work of breathing had already necessitated ongoing high-flow oxygen support. Despite her tachypnea and overall discomfort, she continued to experience periods of lucidity and mental clarity, which allowed her to voice her wishes and concerns in her care.

During these moments, the patient made it known that her greatest priority was spending meaningful time with her husband and 10-year-old son. In an effort to maximize this request, the palliative medicine team began to de-escalate her medication regimen to mitigate the effects of earlier polypharmacy on her overall mentation. Her PCA dose was decreased, with dosing intervals liberalized, and her adjuvant medications were also de-escalated due to perceived minimal benefit as she neared end of life.

Over the next 36 hours, there was improvement in her mentation, and conversations about her current clinical condition were able to be held with her active participation. She was agreeable to enlisting the support of the certified child life specialist and was able to engage in legacy-making activities with her son and husband, while still experiencing a relative improvement in her pain control. Moreover, lengthy bedside discussions were held in conjunction with the patient, family, primary medical service, and palliative medicine to begin to discuss her end-of-life wishes and potential transition to hospice services with comfort care.

Unfortunately, she developed an acute clinical decline the evening after our initial discussions surrounding her transition to hospice and experienced a “code blue” before the palliative medicine team was able to return to confirm code status. She was briefly coded before the husband requested the code to be stopped to allow her to pass peacefully.

The aftermath

Despite this unfortunate conclusion, the palliative medicine team remained actively engaged and was in a unique position to provide ongoing support to the patient’s husband and young son during this vulnerable time of acute grief. Our team provided care not traditionally identified by other medical teams. For example, following her death, the palliative medicine nurse practitioner arranged for transfer of the patient’s body to an institutional research facility. Because of the earlier goals of care discussions, it was known that the patient and family felt strongly about her desire to contribute to the ongoing research of this ultra-rare illness. It was of utmost importance that upon her death, her body would be utilized to advance the understanding of Degos disease with the hope of saving lives in the future.

In addition, several weeks after the patient's death, the child life specialist and palliative medicine physician met again with the patient’s husband and son to allow space to decompress and grieve further. Because of the whole-person care provided to the patient in her last days, and the rapport established in moments of great despair, there was a level of connection and trust that was unmatched in comparison to other teams of providers.

## Discussion

This case of a 38-year-old female diagnosed with systemic Kohlmeier-Degos disease underscores the importance of early involvement of palliative medicine in rare and complex diseases, which can significantly augment both patient care and family support throughout a challenging and lengthy clinical course. An earlier 2025 study in Norway examined the utilization of palliative medicine in children and adults with inherited metabolic diseases throughout Europe. Many of these patients had multi-systemic conditions without curative options. They found that, over the course of the previous five years, 60% of physicians had referred only 20% or fewer of their deceased patients to palliative care [7]. This clearly demonstrates the underutilization of palliative services in vulnerable populations with multi-systemic illness, similar to our patient with Kohlmeier-Degos disease.

The 2013 Shire Rare Disease Impact Report highlighted that individuals with rare diseases and their caregivers experience significant depression, anxiety, stress, isolation, and worry based on future outlook and lack of information [6]. Kohlmeier-Degos disease presents with multisystem vaso-occlusion events, culminating in symptoms such as painful skin lesions, abdominal pain, diarrhea, and neurologic sequelae, among other potential system-specific symptoms. By engaging palliative care earlier in the clinical course, perhaps at the onset of known systemic involvement, the multidisciplinary team can proactively address complex symptom burdens, including pain, nausea, and potential mood disorders that impact day-to-day life.

Although survival in systemic Kohlmeier-Degos disease typically spans two to three years after systemic involvement [8], early palliative care involvement ensures that goals of care, code status preferences, and advance directives are discussed with full transparency, while prognosis is still uncertain, often early in the disease course. In turn, this fosters patient autonomy, realistic expectations, and tailored care paths as new organ involvement appears with the progression of the disease. When there is no cure for a progressive, life-limiting illness, a palliative care approach prioritizes alleviation of physical suffering, preservation of autonomy and dignity, and support for caregivers [6].

The psychological strain of coping with a rare, potentially fatal disease cannot be overstated. In the United States, health-related quality of life in patients with rare diseases is estimated to be 44% of what it would be if the person were healthy [6]. Moreover, quality of life is even lower in people with rare diseases for which there are no treatment options. Because of the multidisciplinary structure of palliative medicine, patients and families can be provided counseling, respite resources, and spiritual and family support frameworks. These support structures help mitigate caregiver burnout and prevent decision-making fatigue, since caregivers also experience decreased quality of life due to high levels of physical strain in caring for those with rare illnesses [6].

Though research on the involvement of palliative care in rare diseases is limited, it is clear that great opportunities exist for early integration in this population. With palliative medicine engagement shortly after diagnosis, this can lead to more overall clarity and meaningful time spent with loved ones throughout the entire disease course.

## Conclusions

The early integration of palliative medicine in systemic Kohlmeier-Degos disease and other rare diseases delivers multidimensional value to patients and families. Symptom management, shared decision-making, complex care coordination, and psychosocial support can all be enhanced by engaging the multidisciplinary expertise that comes with palliative medicine involvement. While early palliative medicine involvement is now better recognized in conditions such as heart failure, lung disease, and metastatic cancers, it is still not commonplace and often overlooked in those patients with rare diseases. For providers and institutions managing patients with rare and life-limiting conditions like Kohlmeier-Degos disease, embedding palliative care at the time of diagnosis, not only near the end of life, should be strongly considered as a pillar of best practice. Though limited research is available evaluating the utilization of palliative care in rare illnesses, this population of patients deserves the same high-quality end-of-life support and palliative care support throughout the duration of their illness.
